# Processing Properties and Potency of *Bacillus thuringiensis* Cry Toxins in the Rice Leaffolder *Cnaphalocrocis medinalis* (Guenée)

**DOI:** 10.3390/toxins15040275

**Published:** 2023-04-06

**Authors:** Yajun Yang, Zhihong Wu, Xiaochan He, Hongxing Xu, Zhongxian Lu

**Affiliations:** 1 State Key Laboratory for Managing Biotic and Chemical Threats to the Quality and Safety of Agro-Products, Institute of Plant Protection and Microbiology, Zhejiang Academy of Agricultural Sciences, Hangzhou 310021, China; 2 Jinhua Academy of Agricultural Sciences, Jinhua 321000, China

**Keywords:** Cry toxin, midgut extracts, *Cnaphalocrocis medinalis*, digestion, potency

## Abstract

Different Cry toxins derived from *Bacillus thuringiensis* (Bt) possess different insecticidal spectra, whereas insects show variations in their susceptibilities to different Cry toxins. Degradation of Cry toxins by insect midgut extracts was involved in the action of toxins. In this study, we explored the processing patterns of different Cry toxins in *Cnaphalocrocis medinalis* (Lepidoptera: Crambidae) midgut extracts and evaluated the impact of Cry toxins degradation on their potency against *C. medinalis* to better understand the function of midgut extracts in the action of different Cry toxins. The results indicated that Cry1Ac, Cry1Aa, and Cry1C toxins could be degraded by *C. medinalis* midgut extracts, and degradation of Cry toxins by midgut extracts differed among time or concentration effects. Bioassays demonstrated that the toxicity of Cry1Ac, Cry1Aa, and Cry1C toxins decreased after digestion by midgut extracts of *C. medinalis*. Our findings in this study suggested that midgut extracts play an important role in the action of Cry toxins against *C. medinalis*, and the degradation of Cry toxins by *C. medinalis* midgut extracts could reduce their toxicities to *C. medinalis*. They will provide insights into the action of Cry toxins and the application of Cry toxins in *C. medinalis* management in paddy fields.

## 1. Introduction

*Bacillus thuringiensis* (Bacillales: Bacillaceae; [Bt]) is a Gram-positive bacterium that generates parasporal crystals (formed mainly by Cry and Cyt proteins), which are activated after solubilization and enzymatic digestion and have insecticidal activity against a variety of insects [1,2,3]. It has been widely used in pest management for several decades due to its insecticidal activity [4,5,6,7]. Upon ingestion by insect larvae, Cry proteins are solubilized and proteolyzed into activated toxins in the alkaline environment of the midgut [8,9]. The activated toxin binds to the receptor on the brush border membrane vesicles (BBMVs); then, an oligomer of the toxin forms, binds with the other receptors on the BBMVs, and is inserted into the membrane, resulting in the formation of pores [10,11], or signal transduction involving Ac/PKA is induced, leading to subsequent cell death [12,13,14,15].

The midgut, an essential insect organ with various proteases, is important in food digestion, utilization, and detoxification [16,17]. Enzymes in midgut juices were reported in the mechanism of Bt action [18]. Cry protoxins are generally processed in the midgut fluids of lepidopteran larvae from 130–140 kDa to 60–70 kDa [19]. However, the activated toxins are digested into smaller molecules and may even be fully destroyed after prolonged contact between toxins and midgut extracts. The digestive activity of insect pests is critical to toxin action and influences toxin toxicity and specificity. When midgut extracts interacted with Cry proteins, two consequences were observed: activation and degradation, which might result in two different toxicity effects [20]. Variations in midgut juices between susceptible and resistant *Plutella xylostella* (Linnaeus) (Lepidoptera: Plutellidae) strains may play an important role in *P. xylostella* resistance [21]. The activated toxin’s stability was shown to be proportional to its toxicity against the target insect [22]. *Anomala cuprea* (Hope) (Coleoptera: Scarabaeidae) neat gut juice demonstrated the capacity to breakdown Cry toxin into smaller, atoxic particles in one minute [23]. Brunet et al. [24] proposed that *Manduca sexta* (Linnaeus) (Lepidoptera: Sphingidae) midgut juice contains protease inhibitors, which may have an essential function in Bt toxin action. Accelerated Cry toxin degradation leads to loss of Cry1C vulnerability in fifth-instar larvae of *Spodoptera littoralis* (Boisduval) (Lepidoptera: Noctuidae) [25], while serine protease inhibitors can increase the insecticidal efficacy of some Cry proteins by up to 20-fold [26].

Rice, *Oryza sativa* L. (Poales: Poaceae), is one of the world’s most important essential foodstuffs [27,28]. It is consumed by roughly half of the world’s population, the majority of whom live in Asia, the primary rice-producing region [28,29]. With the growing human population and consumption, it is critical to enhance the current rice yields [30]. However, rice can be damaged by insect pests, leading to losses in rice yields [31]. *Cnaphalocrocis medinalis* (Guenée) (Lepidoptera: Pyralidae), a significant rice insect pest, is widely distributed in China, Japan, Korea, India, Vietnam, Thailand, the Philippines, Malaysia, and other Asian countries [32,33]. It causes chlorophyll loss by folding and feeding leaves [32,33], and its heavy outbreaks could cause significant losses in rice production [34]. In the worst cases, this pest can cause 63–80% rice yield losses [35]. In 2015, *C. medinalis* damaged 15.5 million ha of rice plants, resulting in yield losses of 0.47 million tons in China [34]. For a long time, the control of *C. medinalis* relied on chemical insecticides. However, the overuse and misuse of chemicals can cause many negative issues, such as environmental pollution and insect resistance. Recently, with the proposal of “reductions in chemicals” in China, an increasing number of nonchemical measures were recommended for pest control in paddy fields. Bt sprays have been used as biological pesticides to control *C. medinalis* for decades [36]. Bt rice cultivars on trial could suppress the population of Lepidoptera insect pests such as *C. medinalis* and mitigate their damage in paddy fields [29]. Several Cry toxins and Bt rice lines have both been shown to be effective against *C. medinalis* [37,38,39]. However, insect tolerance and resistance to Bt toxins may impede their implementation, and many insects were found to be resistant to Bt toxins in laboratory or field populations [40,41,42,43]. A report from Wu et al. [44] suggested that *C. medinalis* has the potential to develop resistance to low amounts of Cry toxin. It is crucial to understand the interaction between Cry toxins and *C. medinalis*. The midgut is an essential organ in which the Cry toxin functions, and midgut extracts play a role in its activity [7,45]. Yang et al. [46,47] reported that pH and inhibitors could influence the protease profiles and the degradation of activated Cry toxins in the midgut juices of *C. medinalis*. Moreover, variations in the toxicities of Cry toxins were observed in *C. medinalis* [38]. Degradation of Cry toxins by insect midgut extracts might be involved in the action of toxins in *C. medinalis*. In this work, we explored the processing patterns of different Cry-activated toxins in *C. medinalis* midgut extracts and evaluated the impact of Cry toxins digestion on their efficacy against *C. medinalis* to better understand the function of midgut extracts in the action of different Cry toxins. Our findings may help to explain variations in the potency of Cry toxins against *C. medinalis*, as well as their interaction with Cry toxins, and will enhance the safe use of Cry toxins.

## 2. Results

### 2.1. Processing of Cry Toxins with Different Concentrations of C. medinalis Midgut Extracts

**Figure 1:**
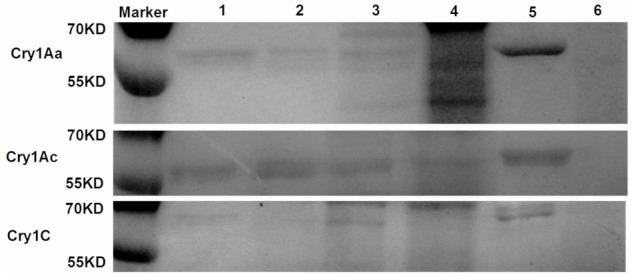
In vitro processing of Cry toxins with different concentrations of *C. medinalis* midgut extracts. For in vitro processing, Cry1Aa, Cry1Ac, and Cry1C (1 μg) were incubated with *C. medinalis* midgut extracts at 30 °C for 8 h at ratios (Cry toxin:extracts, *w*/*w*) of 10:1, 1:1, 1:10, and 1:100, respectively. Cry toxins and midgut extracts were incubated at 30 °C for 8 h as controls, respectively. Processed Cry toxins, after incubation with midgut extracts, were separated by SDS–PAGE gel. Lanes 1–4: 10:1, 1:1, 1:10, and 1:100; Lane 5: Cry toxin; Lane 6: midgut extracts.

Three Cry-activated toxins, Cry1Aa, Cry1Ac, and Cry1C, were processed in *C. medinalis* midgut extracts at four different ratios (10:1, 1:1, 1:10, and 1:100) (Cry toxin:extracts, *w*/*w*) at 30 °C for 8 h, respectively. The results demonstrated that all three Cry toxins in this study could be degraded by *C. medinalis* midgut extracts; however, the degradation levels varied among the Cry toxins. Cry1Aa degradation levels rose with increasing concentrations of *C. medinalis* midgut extracts, and Cry1Aa toxins were entirely degraded into small fragments at a ratio of 1:100 (Cry toxin:extracts, *w*/*w*) (Figure 1). The Cry1Ac and Cry1C toxins showed a similar pattern of degradation as Cry1Aa, whereas Cry1Ac was not totally degraded at a ratio of 1:100 (Cry toxin:extracts, *w*/*w*) (Figure 1).

### 2.2. Processing of Cry Toxins with C. medinalis Midgut Extracts over Time

**Figure 2:**
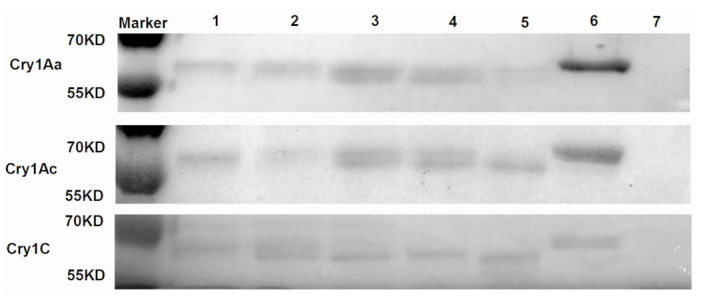
In vitro processing of Cry toxins with *C. medinalis* midgut extracts over various times. Cry1Aa, Cry1Ac, and Cry1C (10 μg) toxins were incubated with *C. medinalis* midgut extracts (1:10, Cry toxin:extracts, *w*/*w*) at 30 °C for 2, 4, 8, 12, and 24 h, respectively. Cry toxin and midgut extracts were incubated at 30 °C for 8 h as controls, respectively. Processed Cry toxins, after incubation with midgut extracts, were separated by SDS–PAGE gel. Lanes 1–5: 2, 4, 8, 12, 24 h; Lane 6: Cry toxin; Lane 7: midgut extracts.

Three Cry-activated toxins, Cry1Aa, Cry1Ac, and Cry1C, were processed in *C. medinalis* midgut extracts (1:10, Cry toxin:extracts, *w*/*w*) at 30 °C over various times (2, 4, 8, 12, and 24 h). The results revealed that when the incubation period was prolonged, the degradation levels of Cry toxins rose. The Cry1Ac, Cry1Aa, and Cry1C toxins began to break down into small fragments after four hours of incubation (Figure 2). After 24 h of incubation, Cry1Ac and Cry1C toxins were entirely degraded into small fragments; however, Cry1Aa was not completely degraded, but the small fragments were further degraded (Figure 2).

### 2.3. Potency of Cry Toxin Processed by C. medinalis Midgut Extracts

**Figure 3:**
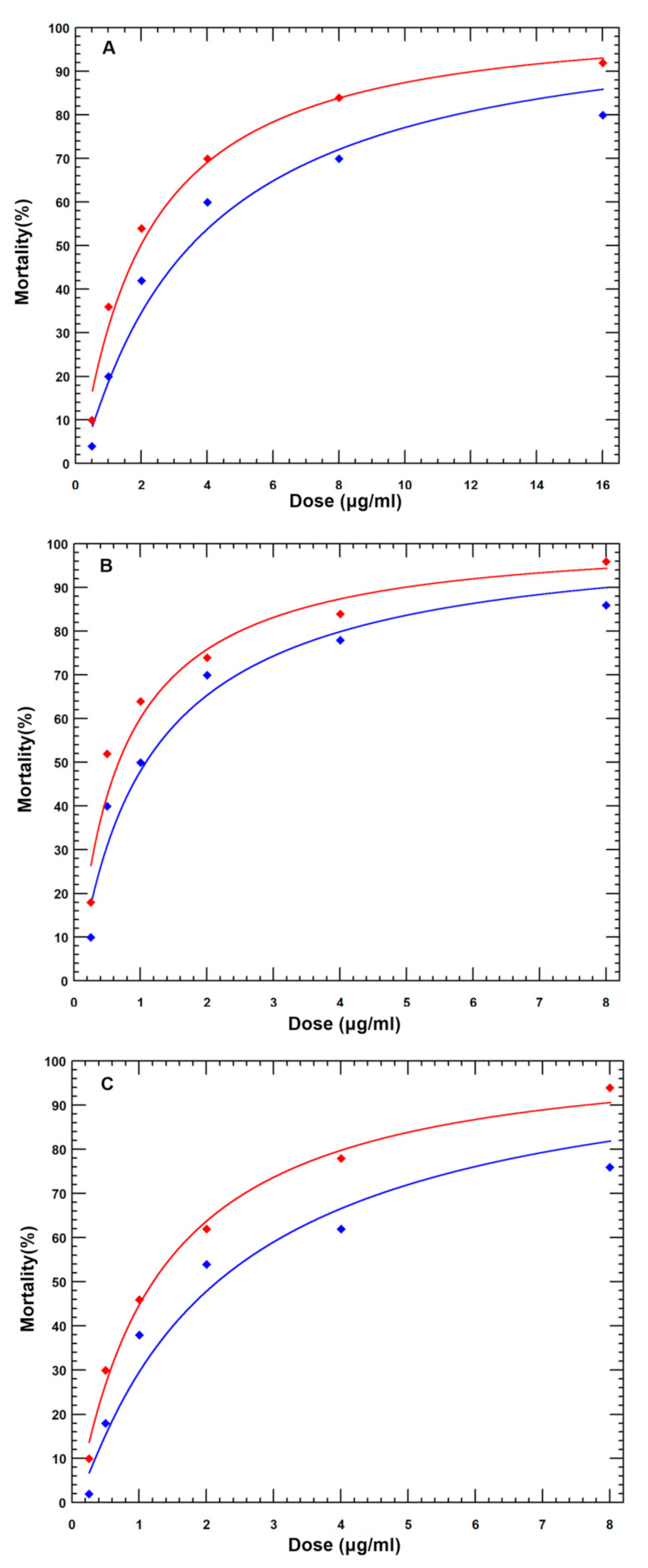
Equality and parallelism of the toxicities of Cry toxins compared with those of Cry toxins processed by midgut extracts against *C. medinalis*. Red and blue lines represent the treatments of Cry toxins and Cry toxins processed by midgut extracts, respectively. (**A**) Cry1Aa toxins with or without midgut extracts digestion (equality: chi-square = 12.39, *p* < 0.05; parallelism: chi-square = 0.24, *p* > 0.05); (**B**) Cry1Ac toxins with or without midgut extracts digestion (equality: chi-square: 7.24, *p* < 0.05; parallelism: chi-square = 0.12, *p* > 0.05); (**C**) Cry1C toxins with or without midgut extracts digestion (equality: chi-square = 12.92, *p* < 0.05; parallelism: chi-square = 0.62, *p* > 0.05).

The toxicities of Cry1Aa, Cry1Ac, and Cry1C toxins, with and without digestion by *C. medinalis* midgut extracts, were evaluated in *C. medinalis* larvae through detached leaf-dipping methods. The Cry-activated toxins processed by midgut extracts were prepared by incubating activated Cry toxin and *C. medinalis* midgut extracts at 30 °C for 8 h at a ratio of 1:10 (*w*/*w*). The LC_50_ values of activated Cry1Aa, Cry1Ac, and Cry1C toxins against *C. medinalis* were 1.981, 0.673, and 1.207 μg/mL, respectively (Table 1). The LC_50_ values of activated Cry1Aa, Cry1Ac, and Cry1C toxins processed by midgut extracts against *C. medinalis* were 3.498, 1.068, and 2.186 μg/mL, respectively (Table 1). After being processed by midgut extracts, the toxicities of activated Cry1Aa, Cry1Ac, and Cry1C toxins decreased. The toxicity regression lines of activated Cry toxins were not equal but rather parallel with those of activated Cry toxins digested by midgut extracts (Figure 3). The ratios of the LC_50_ values of activated Cry toxins processed by midgut extracts to activated Cry toxins varied from 1.586 to 1.811.

## 3. Discussion

Over 200 distinct Cry toxins, derived from Bt, were identified, with the majority being harmful to insect pests and nematodes [48,49]. Numerous studies have revealed differences in the toxicities of Cry toxins [2,36,50,51,52,53,54,55]. The difference in toxicity is affected by many factors, and predigestion treatment by solubilization or enzymatic processing has a great effect [2]. Degradation of Cry toxins by insect midgut extracts might be involved in the action of toxins, and accelerated Cry toxin degradation could reduce or eliminate insecticidal activity [25]. In this study, we explored the degradation properties of Cry toxins in *C. medinalis* midgut extracts and evaluated the potency of the Cry toxins processed by the midgut extracts. All three activated Cry toxins could be degraded by midgut extracts in *C. medinalis,* and the digestion of Cry toxins by midgut extracts could reduce their toxicities against *C. medinalis*. Our findings will help researchers better understand the variations in the toxicity of Cry toxins against *C. medinalis* and aid in the investigation of interactions between *C. medinalis* and the Cry protein.

In insects, the midgut is an essential organ for metabolism and food usage, and enzymes in the midgut play a critical role in these functions [16,17]. Midgut extracts are rich in enzymes that can activate the Cry protein as well as degrade it into smaller peptides [20]. Our results suggested that all three Cry toxins in this study could be degraded by *C. medinalis* midgut extracts, and they began to break down into small fragments after four hours of incubation. However, the degradation levels varied among the Cry toxins. At a ratio of 1:100 (Cry toxin:extracts, *w*/*w*), Cry1Aa and Cry1C toxins were entirely degraded into smaller fragments, whereas Cry1Ac was not totally degraded at a ratio of 1:100 (Cry toxin:extracts, *w*/*w*). After 24 h of incubation, Cry1Ac and Cry1C toxins were entirely degraded into small fragments; however, Cry1Aa was not completely degraded, but the small fragments were further degraded. Tomimoto et al. reported that pronase in *Bombyx mori* (Lepidoptera: Bombycidae) could degrade Cry protein into several tiny fragments [56]. Cry1Ca toxin is completely destroyed when incubated with midgut extracts from high larval instars of *S. littoralis* (Boisduval) (Lepidoptera: Noctuidae) [25]. Cry3Aa toxin is digested into smaller pieces than the 55-kDa activated fragments in the red palm weevil, *Rhynchophorus ferrugineus* (Coleoptera: Curculionidae), under distinct conditions [57]. However, no degradation of any of the toxins was observed in the proteolytic processing of Bt toxins Cry3Bb1 and Cry34Ab1/Cry35Ab1 by western corn rootworm midgut extracts [58]. Our previous studies indicated that pH and inhibitors could influence the degradation of Cry toxins in *C. medinalis* [46,47]. Apart from these factors, protease activity, developmental stages of insects, and the structure of Cry toxins may be connected to Cry toxin degradation in midgut extracts.

Toxicity variations were found in many Cry toxins against *C. medinalis* [38]. We examined the efficacy of Cry toxins against *C. medinalis* with and without digestion by midgut extracts in this study. The results showed that a Cry toxin digested by *C. medinalis* midgut extracts had reduced toxicity. A protein complex in the midgut of the spruce budworm, *Choristoneura occidentalis* Freeman (Lepidoptera: Tortricidae), might inactivate the Cry toxin by precipitation [59]. Previous studies indicated that overdigestion of Cry toxins by lepidopteron midgut juice was normally associated with a loss of toxicity [20,60,61]. Pang et al. [62] discovered that an increase in midgut juice content was associated with a reduction in the insecticidal efficacy of Cry toxin due to the generation of nonactive pieces. Smaller fragments of Cry1Ab toxin degraded by midgut extracts of *M. sexta* and *Spodoptera frugiperda* (Lepidoptera: Noctuidae) were correlated with a decrease in pore formation and insecticidal activities, and cleavage in domain II of Cry1Ab toxin may be involved in toxin inactivation [20]. The toxicity of *B. thuringiensis* var. *thuringiensis* to *Pieris brassicae* (Lepidoptera: Pieridae) and *Mamestra brassicae* (Lepidoptera: Noctuidae) was shown to be closely linked to protein content and activity in the midgut [63]. Moreover, in resistant strains of insects, decreased toxicity was associated with alterations in midgut juice [20,61,64]. The initial stage influencing the variations in the toxicity of Cry toxins was the distinct digestive levels of midgut juice for various Cry toxins. Brunet et al. [24] proposed that *M. sexta* midgut fluid components might influence pore formation by Cry9Ca toxin. Yamazaki et al. [21] discovered that midgut extracts of *P. xylostella* (Lepidoptera: Plutellidae), which are highly resistant to Cry1Ac, possess three times larger amounts of glucosinolate sulfatase, which binds to Cry1Ac, compared to susceptible strains. Tetreau et al. [65] found that midgut extract alterations were involved in the process of Bt resistance in the yellow fever mosquito *Aedes aegypti* (Linnaeus) (Diptera: Culicidae) using proteomic and transcriptomic approaches. Changes in the enzymes in the midgut juice also influence Cry1Ac toxicity against *Heliothis virenscens* (Fabricius) (Lepidoptera: Noctuidae) through a proteinase inhibition assay [66]. Engineering multiple trypsin/chymotrypsin sites in the Cry3A toxin could enhance its activity against *Monochamus alternatus* (Coleoptera: Cerambycidae) larvae [67].

Cry toxin activity was linked not only to midgut enzymes but also to a series of receptors on the BBMV in the midgut [68]. Karim and Dean [39] found that Cry1Ac, Cry1Ab, and Cry1Aa had distinct high binding affinities to *C. medinalis* and were linked to an essential step in the Cry toxin’s action. Yang et al. [69] discovered that several genes may be implicated in the *C. medinalis* reaction with the Cry toxin. Recently, at least seven ABC proteins were reported to be associated with the *C. medinalis’* response to the Cry1C toxin [70]. Our results indicated the importance of midgut extracts in the degradation of Cry toxins. Interestingly, *C. medinalis* possesses the potential to develop resistance to a low amount of Cry toxin by increasing the activities of the main enzymes in the midgut [44]. Prevention of the further degradation of Cry-activated toxins might maintain their toxicity against *C. medinalis*. In the case of *Ephestia kuehniella* (Lepidoptera: Phycitidae), Cry1Ac toxicity was enhanced toward this lepidopteran pest through the toxin’s protection against excessive proteolysis [71]. As an important material for bioagents, Cry toxins play a crucial role in sustainable agriculture. Delaying insect resistance or maintaining the toxicity of Cry toxin is important for the application of Bt toxins. The findings in our study provide novel insights into the potential threat of *C. medinalis* resistance to Cry toxins and promote the development of sustainable agriculture. Our results in this research only provide one perspective on the interplay of Cry toxins and *C. medinalis* via Cry toxin digestion. More investigations on the interaction of Cry toxins and *C. medinalis* will elucidate the mechanism of Cry toxins in *C. medinalis*, boosting the application of Cry toxins in *C. medinalis* management.

## 4. Conclusions

Herein, we investigated the degradation properties of Cry toxins in *C. medinalis* midgut extracts and tested the efficacy of the Cry toxins processed by the midgut extracts. The results suggested that midgut extracts from *C. medinalis* could degrade the activated Cry toxins, and the degradation levels of Cry toxins by midgut extracts differed depending on the time or concentration effects. In addition, further degradation of Cry toxins by midgut extracts could reduce their toxicities to *C. medinalis.* The findings here will facilitate the understanding of Bt action on *C. medinalis* and promote Bt application in the *C. medianlis* control.

## 5. Materials and Methods

### 5.1. Insects

*C. medinalis* adults were gathered using a sweep net from paddy fields (30.7° N, 120.9° E) in Jiaxing, Zhejiang, China. The moths were given a 10% honey solution in a plastic cup covered with nylon mesh. Eggs laid on the mesh were removed and placed in a box with a detached leaf from a 45-day-old Taichung Native 1 (TN1) rice plant. The insect cultures were maintained at 27 ± 1 °C with a relative humidity of 70–80% and a photoperiod of 14:10 (L:D) h.

### 5.2. Toxins

Activated Cry1Ac, Cry1C, and Cry1Aa toxins (MP, Cavey, CWRU, US) derived from *Bacillus thurigensis* were purchased from Youlong BioTech Co., Ltd. (Shanghai, China). They were produced from Cry protoxins through proteolysis using trypsin and refined using ion exchange HPLC, and the activated Cry toxins were really 97% pure.

### 5.3. Preparation of Midgut Extracts

*C. medinalis* fifth-instar larvae were cooled on ice for 30 min before their midgut tissues were dissected. The midgut fluids were separated from the solids by centrifugation at 10,000× *g* for 20 min and then filtered through 0.22-m filters. The total protein content of midgut extracts was measured through Bradford’s method [72] with a microplate reader (Tecan Trading AG, Mannedorf, Switzerland). The midgut extracts were aliquoted and kept at −70 °C until needed.

### 5.4. Processing of Cry Toxins with Different Concentrations of C. medinalis Midgut Extracts

One microgram of activated Cry toxin was combined with midgut extracts at various ratios (Cry toxin:midgut extracts, *w*/*w*: 10:1, 1:1, 1:10, and 1:100) and incubated at 30 °C for 8 h. Toxin digestion was halted using a 1 mM solution of phenylmethylsulfonyl fluoride (PMSF, Sigma-Aldrich^®^, Sigma Aldrich (Shanghai) Trading Co., Ltd., Shanghai, China). 

Protein was separated using an 8–10% SDS-PAGE and stained with Coomassie brilliant blue.

### 5.5. Processing of Cry Toxins with C. medinalis Midgut Extracts over Time

In vitro testing of Cry toxin degradation levels by midgut extracts over time was performed. Ten micrograms of activated Cry toxins were incubated at 30 °C with midgut extracts at a concentration of 1:10 (Cry toxin:midgut extracts, *w*/*w*) for 2, 4, 8, 12, and 24 h, respectively. Toxin digestion was halted by 1 mM PMSF. Protein was separated using an 8–10% SDS-PAGE and stained with Coomassie brilliant blue.

### 5.6. Bioassays

Activated Cry toxins and Cry toxins processed by midgut extracts were employed to determine insecticidal activity against *C. medinalis* larvae. The Cry-activated toxins processed by midgut extracts were prepared by incubating activated Cry toxin and *C. medinalis* midgut extracts at 30 °C for 8 h at a ratio of 1:10 (*w*/*w*). The bioassays were delivered using the detached leaf-dip technique with modifications [73]. A final 0.1% Triton X-100 solution was prepared in solutions for diluting and spreading over the rice leaf. The leaves of the main rice stalks were chopped into 3–4 cm portions and soaked in every solution for 1 min before being placed in Petri dishes coated with damp absorbent cotton (5 cm in diameter). 0.01 M PBS (containing 0.1% Triton X-100) was used to treat control leaves. Ten second-instar larvae were placed into each Petri dish using a camel hair brush, and the dishes were then sealed with Parafilm^®^ (Sigma Aldrich (Shanghai) Trading Co., Ltd., Shanghai, China) to keep the larvae from escaping and covered with wet mesh to keep the moisture in. Each treatment was carried out five times. After 48 h, the survival rate was calculated.

### 5.7. Data analysis

Bioassay data were analyzed through probit analysis using POLO Plus software (version 2.0) (LeOra Software, Berkeley, CA, USA), with a natural response (control mortality) included as a model parameter, and the equality and parallelism analysis of probit-regression lines were also treated with POLO Plus software [74,75].

## Figures and Tables

**Table 1:** Median lethal concentrations of Cry toxins and Cry toxins processed by midgut extracts against *C. medinalis*.

Toxins	*n*	Slope (SE)	LC_50_ (95%FL) (μg/mL)
Cry1Aa	300	1.684 (0.181)	1.981 (1.556–2.472)
Cry1Aa processed by midgut extracts	300	1.562 (0.174)	3.498 (2.438–5.136)
Cry1Ac	300	1.511 (0.180)	0.673 (0.397–0.989)
Cry1Ac processed by midgut extracts	300	1.427 (0.168)	1.068 (0.664–1.609)
Cry1C	300	1.695 (0.180)	1.207 (0.961–1.506)
Cry1C processed by midgut extracts	300	1.498 (0.174)	2.186 (1.463–3.534)

*n* = number of larvae in the probit analysis. LC_50_ (median lethal concentration): concentration of toxins (µg/mL) required to kill 50% of larvae over 48 h. 95% FL = 95% fiducial limits.

## Data Availability

Not applicable.
